# Translational profile of coding and non-coding RNAs revealed by genome wide profiling of ribosome footprints in grapevine

**DOI:** 10.3389/fpls.2023.1097846

**Published:** 2023-02-08

**Authors:** Zhang Zhen, Fan Dongying, Song Yue, Zhang Lipeng, Liu Jingjing, Liu Minying, Xu Yuanyuan, He Juan, Song Shiren, Ren Yi, Han Bin, Ma Chao

**Affiliations:** ^1^ Shanghai Collaborative Innovation Center of Agri-Seeds, School of Agriculture and Biology, Shanghai Jiao Tong University, Shanghai, China; ^2^ Department of Horticulture, College of Agriculture, Shihezi University, Shihezi, Xinjiang, China; ^3^ Changli Research Institute of Fruit Trees, Hebei Academy of Agricultural and Forestry Sciences, Changli, Hebei, China

**Keywords:** grape, Ribo-seq, DNA JA6, Hsp70, heat stress

## Abstract

Translation is a crucial process during plant growth and morphogenesis. In grapevine (*Vitis vinifera* L.), many transcripts can be detected by RNA sequencing; however, their translational regulation is still largely unknown, and a great number of translation products have not yet been identified. Here, ribosome footprint sequencing was carried out to reveal the translational profile of RNAs in grapevine. A total of 8291 detected transcripts were divided into four parts, including the coding, untranslated regions (UTR), intron, and intergenic regions, and the 26 nt ribosome-protected fragments (RPFs) showed a 3 nt periodic distribution. Furthermore, the predicted proteins were identified and classified by GO analysis. More importantly, 7 heat shock-binding proteins were found to be involved in molecular chaperone DNA J families participating in abiotic stress responses. These 7 proteins have different expression patterns in grape tissues; one of them was significantly upregulated by heat stress according to bioinformatics research and was identified as DNA JA6. The subcellular localization results showed that VvDNA JA6 and VvHSP70 were both localized on the cell membrane. Therefore, we speculate that DNA JA6 may interact with HSP70. In addition, overexpression of *VvDNA JA6* and *VvHSP70*, reduced the malondialdehyde (MDA) content, improved the antioxidant enzyme activity of superoxide dismutase (SOD), catalase (CAT) and peroxidase (POD), increased the content of proline, an osmolyte substance, and affected the expression of the high-temperature marker genes *VvHsfB1, VvHsfB2A, VvHsfC* and *VvHSP100*. In summary, our study proved that VvDNA JA6 and the heat shock protein VvHSP70 play a positive role in the response to heat stress. This study lays a foundation for further exploring the balance between gene expression and protein translation in grapevine under heat stress.

## Introduction

Grapevine is an important fruit tree grown worldwide and is popular for its high nutrition and good color, aroma and taste. According to the latest data from 2021 released by the International Organization of Grapes and Wine (OIV), the planting area of grapevine has already reached 783,000 hectares in China (Dixon et al., 2022), and there is high demand for fresh and wine grapes in the fruit market. Therefore, there is a need to improve the yield and quality of grapevines for increased economic benefits (Zhou et al., 2022).

The transcriptome and proteome provide useful information for examining gene or protein abundance in plants. All transcriptome research aims to characterize transcripts expressed within specific tissues or stages involved in plant growth and development (Smythers and Hicks, 2021). For instance, RNA-seq data was used to generate network maps of 343 salt stress-related differentially expressed genes (DEGs) that were related to metabolic pathways, secondary metabolite biosynthesis, membrane transport, and development pathways and helped identify the functions of genes associated with Thompson Seedless grapes (Wang et al., 2016). Since there are many unlabeled transcripts and unannotated genes in the grape genome (Li et al., 2021), the study of gene function cannot be restricted to the nucleic acid level by transcriptome analyses. Even though 164,062 proteins have been recorded in the UniProt database (Kuang et al., 2019), many more unidentified proteins still need to be revealed. Proteins serve as both direct executors and final controllers of gene activity since they are the building blocks of all life activities (Bheri et al., 2021). Proteins are involved in a variety of plant activities, including maturation, apoptosis, stress resistance, differentiation, and proliferation (Desvoyes and Gutierrez, 2020). Understanding the intricate translation mechanism in plants requires revealing the shifting profile of protein abundance. The isolation and identification of protein samples form the foundation of proteomics (Demir et al., 2018). One method that is frequently used to examine proteins at both the quantitative and qualitative levels is liquid chromatography−mass spectrometry (LC−MS) (Wang et al., 2002). SDS−PAGE is also employed to examine total plant proteins that have been extracted (Sawada and Hirai, 2013). However, only 10,000 proteins have been discovered, and it is challenging to capture entire proteins, particularly those with lower molecular weights (Alves et al., 2019). Ribosome profiling (Ribo-seq), a significant technological advancement that uses a translation inhibitor to stop mRNA from being translated *via* ribosomes (Ruiz-Orera and Albà, 2019), adds a nuclease to treat unprotected mRNA, isolates ribosomes, and extracts short, undigested mRNAs (approximately 30 nt) on the ribosome. These RNAs are then reverse transcribed into cDNA for deep sequencing. The combination of mRNA with polysomes helps detect their active translation position (Hsu and Benfey, 2018). Based on this technology, together with a semisupervised method relying on stacking classification cases, 5360 potentially translated open reading frame (ORFs) in 2051 genes were identified. These presumably formed uORFs were affected by the transcription initiation site and alternative splicing events (Hu et al., 2016). Over 90% of the short ORFs in annotated noncoding RNAs are mapped by super resolution ribosome profiling to the mORF. Many of these unannotated ORFs can create stable proteins and are also conserved (Hsu et al., 2016). The prediction of translated small ORFs showed that lncRNAs can produce peptides in the roots of *Arabidopsis thaliana* (Bazin et al., 2017). Ribosome footprint mapping to a genome-wide location can be used to decipher the photomorphogenic translation process. There are two initiator codons, ATG and CTG, but only ATG constitutes the upstream ORF, thus reducing the translation efficiency of downstream ORFs (Liu et al., 2013). In addition, translational diversity was detected by Ribo-seq in *Chlamydomonas*, rice, maize and tobacco in response to ethylene signal transduction (Merchante et al., 2015), immune induction (Xu et al., 2017), sublethal hypoxia stress (Juntawong et al., 2014), heat stress (Lukoszek et al., 2016), and phosphorous (Pi) nutrition-deficient stress (Bazin et al., 2017). As the whole transcriptome translates real-time sequence data, Ribo-seq is developing into a helpful tool for researchers to track the molecular translation of the proteome (Ingolia et al., 2009). It has potential applications in the study of protein translation processes.

Heat stress seriously influences fruit yield and quality, and plants can arrest defense system processes by reducing the damage to membrane lipids and increasing protective enzyme activities and osmotic substance contents, which helps protect themselves against damage. For example, heterologous overexpression of the *ZnJClpB1-C* gene in tobacco reduced the increase in the content of MDA compared to WT (Panzade et al., 2020). At the same time, a large number of reactive oxygen species (ROS) accumulate under heat stress (Slimen et al., 2014; Medina et al., 2021). When plants are exposed to moderately elevated temperatures, the antioxidant enzyme activities of POD (Janda et al., 2019; Mohapatra et al., 2020), catalase (CAT) (Tutar et al., 2017; Wang et al., 2018; Hassan et al., 2020), and SOD (Wu et al., 2020; Khurshid et al., 2021; Liu et al., 2021) increase, which improves the antioxidant defense process and provides protection from oxidative damage (Miao et al., 2020). Additionally, heat stress transcription factors (Hsfs) are conserved throughout the evolution of eukaryotes, and these factors bind to downstream genes encoding transcription factors, enzymes, and chaperone proteins. Under normal conditions, HSP70 and HSP90 interact with the core regulatory factor heat stress transcription factor A1 (*HsfA1*) and inhibit its activity (Yamada et al., 2007). After heat stress, *HsfA1* is released from HSP70/90 and activated to participate in heat stress (Hahn et al., 2011). In *Arabidopsis thaliana*, a member of the DNA J domain chaperone protein named ATDjB1 interacts with HSP70, which plays a positive regulatory role in response to heat stress through maintaining redox homeostasis (Zhou et al., 2012). However, their particular functions and target genes or proteins are still unclear. Thus, in this work, we identified the molecular chaperone protein DNAJ A6, which is significantly induced by heat stress, through Ribo-seq and GO analysis, and we speculated that it may bind to the heat stress protein HSP70. Furthermore, *VvDNAJ A6* and *VvHSP70* overexpressing tobacco plants accumulated more protective enzymes and osmotic substances involved in heat stress. The complicated mechanisms induced by thermoresistance in grapevine were explored at the physiological and molecular levels.

## Materials and methods

### Plant materials

Grape (*Vitis Vinifera* L.) named ‘Thompson Seedless’ as explant, sterilized with 70% absolute ethanol for 30 s, 15% NaClO, stored at 4°C for 24 h, after that repeat sterilized again, use sterile water to rinse for 6-8 times. The 2-3 cm stems were planted on 1/2 MS+30 g/L sucrose+0.8% Agar+0.1 mg/L NAA media. In addition, the day/night (16 h/8 h) temperatures were required to be 25°C. At the same time, we have obtained overexpression grape callus materials, which are currently in the process of identification.

### Abiotic stress treatment

To test the response of *VvDNA JA6* and *VvHSP70* to heat stress, the 5 weeks old grape subcultured seedlings were placed in a growth incubator at 45°C. For cold stress, the seedlings were placed in a growth incubator at 4°C. For drought stress, the seedlings treatment concentration is 200 mM mannitol. For salt stress, the seedlings treatment concentration is 150 mM NaCl. At the same time, WT was cultured at 25 ± 1°C. All of the WT and treated seedlings were collected at 0, 2, 4, 6, and 8 h. Then grapevine leaves were sampled for RNA extraction.

To further observation of tobacco phenotype under heat stress, the constructed vector of 35S::VvDNA JA6-GFP, 35S::VvHSP70-GFP, and 35S::pSuper1300-GFP were transferred into the GV3101 competence cell. Inoculate three kinds of agrobacterium to LB+50 mg/L kanamycin+20 mg/L rifampicin, cultured 16 h at 28°C with 200 rpm shaking. Twice inoculated to 50 mL liquid medium. 4°C 4000 rpm 10 min. Use suspension containing 0.2 M As, 0.5 M MES and 1 M MgCl_2_ adjusted OD600 nm reached at 0.6-0.8. Injected prepared bacterial solution into the back of 4 weeks old tobacco leaves. Dark co-cultured for 12 h at 25°C. Phenotype observation after treatment at 45°C for 8 h.

### Extraction of ribosome and construction of the library

**Figure 1:**
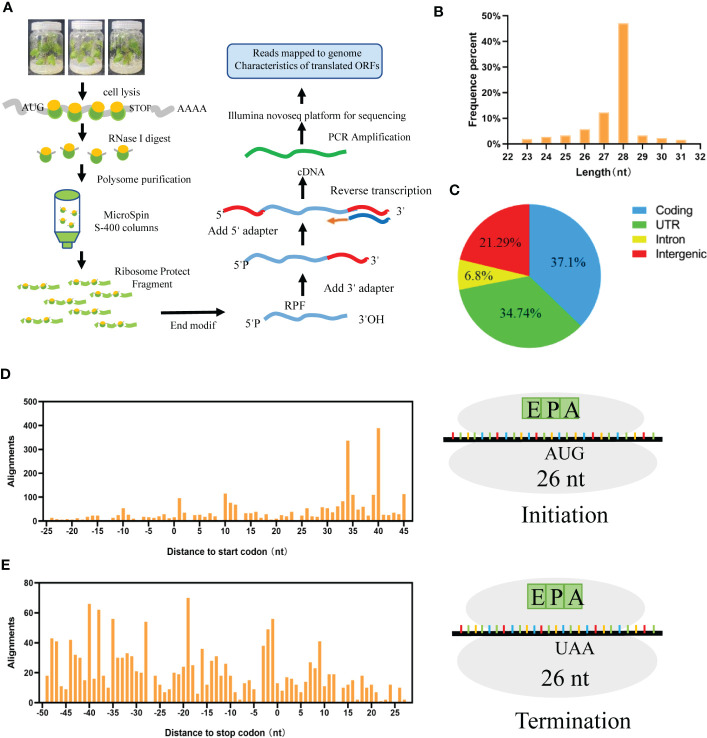
Translation landscape in grapes. **(A)** Overview of the ribosome sequencing approach, the mRNA-tethered ribosomes were extracted and enriched, which performed library preparation and deep sequencing. **(B)** mRNA protected by ribosome fragment enriched at 23-31nt. **(C)** Protein translation regions are distributed in coding, UTR, Intron and Intergenic. **(D)** Ribosomes move in units of 3 nt nucleotides in the initial codon. **(E)** Ribosomes move in units of 3 nt nucleotides in a stop codon.

To reveal the overall translation landscape in grapes, ribosome profiling was performed as Ingolia et al. (2009) described. For the previous approach, we made minimal adjustments: Take 0.1g of tissue and grind it into powder fully, add 1 mL polysome buffer (50 mM Tris-Cl pH 7.5; 0.2 M KCl; 15 mM MgCl_2_) and lysis buffer (10% Triton X-100; 10 µL β-Mercaptoethanol; 10 µL Dnase I; 50 mg/mL cycloheximide), then ribosomes were collected by mircrospin S-400 columns. After the rRNA was removed, it was purified with PAGE gel, the target fragment was screened, and the library containing the fragment was obtained, and then sequenced by Illumina. The detailed flow chart was described in **Figure 1A**.

### Ribo-seq raw data analysis

Remove rRNA, tRNA, adapter and low-quality reads from the raw data to get clean data. Because binary alignment map (BAM) files are compressed binary files, it is hard to check them. So we use the following software to realize visualization: (1) Java download address: (https://www.java.com/zh-CN/); (2) IGV registered address: (https://software.broadinstitute.org/software/igv/?q=registration); (3) IGV download address: [Downloads|IntegrativeGenomicsViewer(broadinstitute.org)]. Secondly, import BAM files and grape genome files (http://plants.ensembl.org/index.html) into IGV. This software can display the distribution of reads on each chromosome, and it also shows the location of sequence on the genome (annotated CDS, introns, intergenic regions and other functional regions).

### Function annotation analysis

Gene ontology (GO) analysis was performed to discover further functional significance enrichment analysis of the screened proteins. The basic unit of GO is the term, and each term corresponds to a gene attribute. Input Gene ID on Omic Share Tools (https://www.omicshare.com/tools/Home/Soft/gogseasenior), then downloads the compressed file for free (**Supplementary Table S1**). Check Molecular Function (MF), Biological Process (BP) and Cellular Component (CC), merge the same item by item in excel.

### Evolutionary tree and domain analysis

Blast VIT_00s0324g00040; VIT_06s0080g01230; VIT_08s0056g01490; VIT_09s0002g07210; VIT_13s0073g00560; VIT_15s0021g02090; VIT_18s0001g14090 protein sequences, choose organism option is *Arabidopsis thaliana*, the highly similar protein sequences were retained. Use MEGA 11 to construct an evolutionary tree, then beautify it with AI software.

All protein sequences were used to construct the evolutionary tree by using the software of Pfam Scan, arranging the txt file in CDD batch search and inputting the protein sequence fasta file on Tbtools software.

### Subcellular localization of DNA JA6 and HSP70

Transient transformation consisted of the method described as phenotype observation. After co-cultured for 3-5 d, then identified protein location using a fluorescence microscope.

### Physiological index detection

The MDA content detection was described as (Li et al., 2018), Take 0.1 g material in 1.8 mL 10% trichloroacetic acid (TCA) solution and put it into the grinding machine for 1 min. 4000 r/min for 10 min. 1 mL supernatant was added to 0.6% thiobarbituric acid (TBA), after boiling water bath for 15 min, Absorbance was checked at 532 nm, 600 nm and 450 nm respectively.

SOD and POD are important antioxidant enzymes in plants, and their activities are closely related to stress resistance. In order to detect activities, 0.1 M/L phosphate buffer (pH 7.6) was added to 0.1 g material and constant volume to 2 mL. Put it into the grinding machine for 1 min. 12000 r/min for 10 min. Then supernatant is the crude extract for protective enzymes. SOD detection was described by (Sattar et al., 2020), and for POD detection, it was described by (Li et al., 2018).

When encountering stress, the content of free proline accumulated greatly. The content of proline detection was described as (Rajametov et al., 2021).

### Yeast two hybrid

Yeast two-hybrid system was used to determine the interaction between VvHSP70 and VvDNA JA6, The full-length VvHSP70 were cloned into the pGBKT7 vector, then transformed into the yeast strain Y2H Gold. Yeast only grew on DO Supplement-Leu/-Trp and did not grow on DO Supplement-Ade-His-Leu-Trp. This indicated that HSP70 has no self-activation effect. Then the full length VvDNA JA6 was cloned into the pGADT7 vector, put VvHSP70-pGBKT7and VvDNA JA6- pGADT7 resulting vectors were transformed into the yeast strain Y2H Gold. pGBKT7and BD-53 resulting vectors as positive control, pGBKT7and BD-Lam resulting vectors as negative control. Observe the growth after a few days.

### qRT-PCR

Extraction of RNA from grape leaves containing polysaccharides and polyphenols by CTAB Method (Chen et al., 2020). Reverse Transcription to cDNA (Fastking RT Kit with gDNase, TIANGEN), uniform RNA loading concentration then add SYBR Green (Talent qPCR Premix, TIANGEN) and quantitative primer (**Supplementary Table S2**). The qRT-PCR conditions were: 95°C 3 min, 40 cycles for 95°C 5 s and 60°C 15 s, Melting for 10 s. Use the method of 2^-ΔΔct^ to calculate the multiple of gene expression change (Trick et al., 2021).

### Statistical analysis

One-way ANOVA variance analysis used the Student’s t-test in GraphPad Prism 8 software to compare the significant difference of the mean between groups, and the significance level was set as α= 0.05 (*, P<0.05; **, P<0.01).

### Accession numbers

Ribo-seq sequencing raw data from this paper can be found in the National Center for Biotechnology Information’s Sequence Read Archive (SRA) under accession number PRJNA903951.

## Results

### Characteristics of ribosome sequencing data from grapevine

To explore the whole translational landscape of all ribosome-protected mRNA regions in grape, Ribo-seq was performed as described by Ingolia (Ingolia et al., 2009). First, mRNA-binding ribosomes were isolated. After that, mRNA segments bound to ribosomes were selected, sequenced, and quantified to create footprints (**Figure 1A**). We obtained 37 million clean data reads, with a Q 30 of 97.09%; ribosome-protected fragments (RPFs) were highly enriched at approximately 23-31 nt (**Figure 1B**). According to the comparison results, the proportions of reads in the coding region, UTR, intron and intergenic region were 37.1%, 34.74%, 6.8% and 21.29%, respectively (**Figure 1C**). During translation, ribosomes move in units of 3 nt along RNA codons to produce translation footprints. Therefore, based on the P site (peptide-based transfer site), RPFs derived from a normal translation process should show a periodic distribution of 3 nt on RNA. The 26 nt RPF occurred 15 nt upstream of the start codon (**Figure 1D**) and disappeared 20 nt downstream of the stop codon (**Figure 1E**).

### Grape mRNA exhibits variable degrees of ribosome binding

RPKM is the unit used to describe the expression efficiency of transcripts. In total, we found that 8291 transcripts were translated in grapes (**Supplementary Table S3**); the top ten most translated proteins are listed in **Table 1**. These proteins have different positions and are related to glutathione S-transferase, photomorphogenesis, transposase and metal combinations. The high expression of these proteins indicated their crucial functions in plant growth and response to biotic and abiotic stresses. In addition to the coding region, we also detected the RPKM values for UTR, intron and intergenic regions (**Supplementary Figure 1**). These results suggest that there were a large number of unconventional peptides that need to be further explored in grapes.

**Table 1:** The higher RPKM among all detected protein.

Number	Accession ID	RPKM	Gene chr	Start	End	Strand	Gene description
1	VIT_07s0031g01060	165548.9298	7	17205450	17206657	+	Glutathione S-transferase
2	VIT_13s0019g02630	145074.2494	13	3884948	3890320	–	Photosystem II protein D1
3	VIT_07s0129g00790	10772.74669	7	16008001	16008525	+	RuBisCO_large domain-containing protein
4	VIT_07s0031g03000	10408.01897	7	19731440	19732022	–	RuBisCO_large domain-containing protein
5	VIT_00s0505g00050	8692.384864	Un	30810787	30810992	–	Transposase
6	VIT_19s0014g02740	5354.452236	19	2789637	2790200	–	Putative metallothionein-like protein
7	VIT_00s0246g00200	4647.555775	Un	17200655	17201478	–	Photosystem I iron-sulfur left
8	VIT_08s0007g00330	4409.562374	8	14633089	14634214	+	Metallothionein
9	VIT_16s0039g00380	4277.243779	16_random	186608	187107	+	Ribosomal protein S14
10	VIT_03s0038g00710	3750.700839	3	614468	614930	+	PSII-J

“+” represent gene is generated by sense strand in the genome."-" represent gene is generated by antisense strand in the genome.

**Figure 2:**
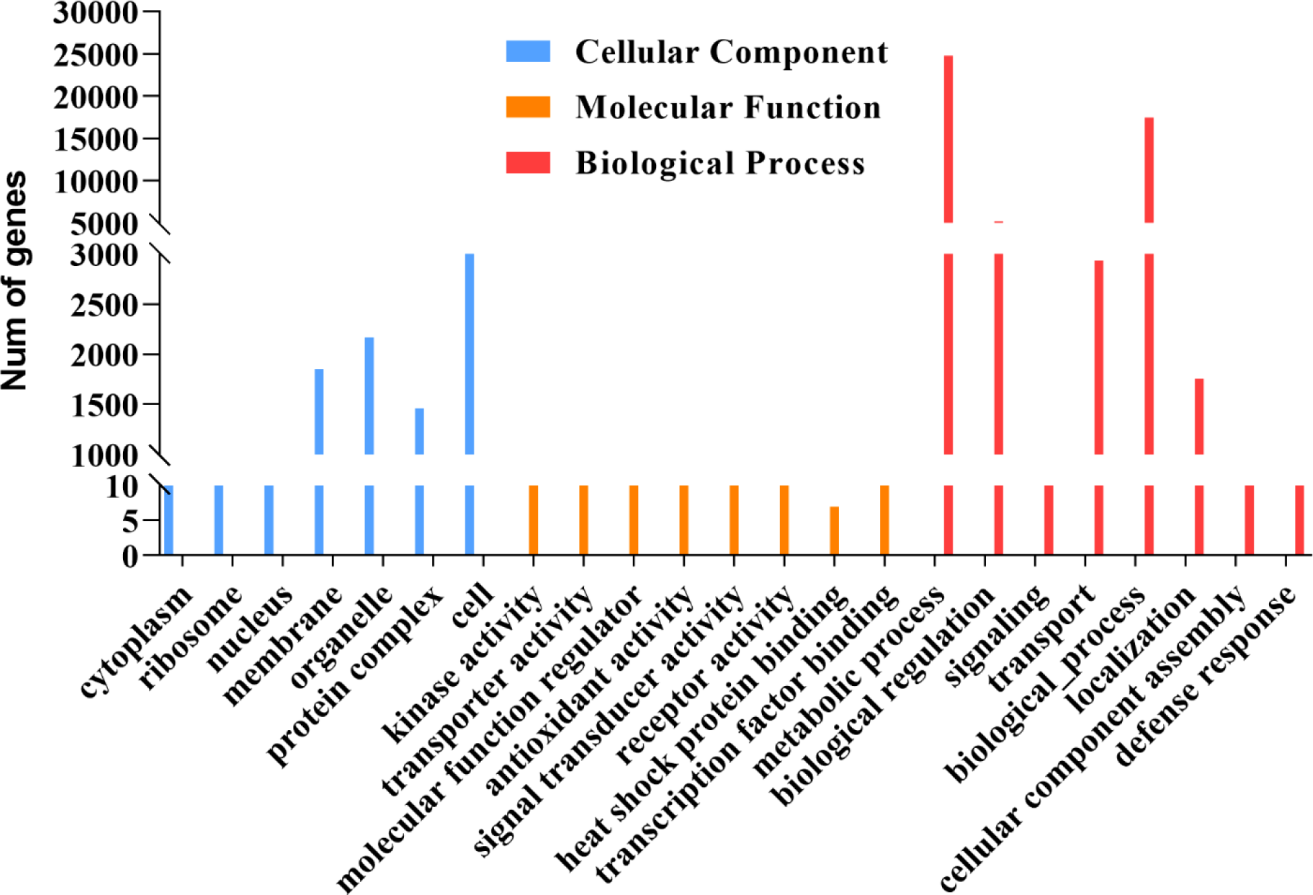
GO secondary function analysis 8291 transcripts are translated in grapes. The blue bar chart represents a cellular component, the orange bar chart represents a molecular function, red bar chart represents the molecular function.

GO secondary function analysis was used to identify the biological role of the 8291 detected proteins. The findings revealed that membrane, organelle, protein complex, and cell proteins had the highest levels of enrichment in the cellular component (CC) category. In terms of the biological process (BP) category, metabolic process, transport, biological process, and regulation were the most enriched GO keywords. Kinase activity, transporter activity, molecular function regulator, antioxidant activity, signal transducer activity, and similar items were found for the molecular function (MP) category. Interestingly, we discovered 7 proteins in the UniProt public database that are involved in heat shock protein binding (VIT 00s0324g00040, VIT 06s0080g01230, VIT 08s0056g01490, VIT 09s0002g07210, VIT 13s0073g00560, VIT 15s0021g02090, and VIT 18s0001g1409) (**Figure 2**).

**Figure 3:**
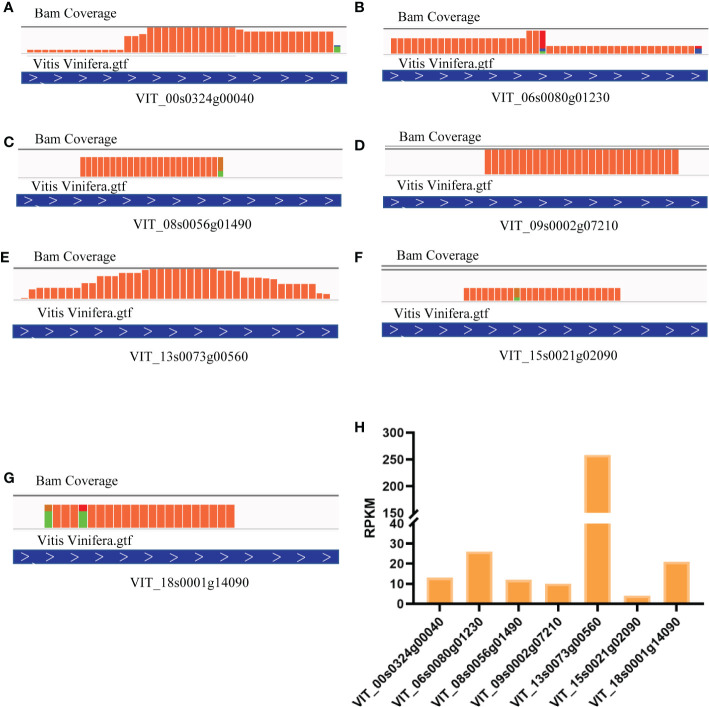
Comparation and visualization of translation efficiency in heat shock binding proteins. **(A)** RPKM in Different heat shock binding proteins. Reads coverage in VIT_00s0324g00040 **(B)**, VIT_06s0080g01230 **(C)**, VIT_08s0056g01490 **(D)**, VIT_09s0002g07210 **(E)**, VIT_13s0073g00560 **(F)**, VIT_15s0021g02090 **(G)**, VIT_18s0001g14090 **(H)**.

These 7 heat shock binding proteins were selected for further research. We activated all ribosome footprints to appraise and compare translation efficiency, and the results showed highly different ribosome binding levels. To visualize the translational activity, we mapped clean reads to the genome of *Vitis vinifera L.* The total read coverage of VIT_00s0324g00040 (**Figure 3A**), VIT_06s0080g01230 (**Figure 3B**), VIT_08s0056g01490 (**Figure 3C**), VIT_09s0002g07210 (**Figure 3D**), VIT_13s0073g00560 (**Figure 3E**), VIT_15s0021g02090 (**Figure 3F**), and VIT_18s0001g14090 (**Figure 3G**) could be observed by using Integrated Genome Viewer (IGV) software. The highest translation efficiency was observed for VIT_13s0073g00560. Conversely, VIT_15s0021g02090 had the lowest translation efficiency in grapevine (**Figure 3H**).

### Heat shock binding proteins play an important role in the plant response to abiotic stresses

**Figure 4:**
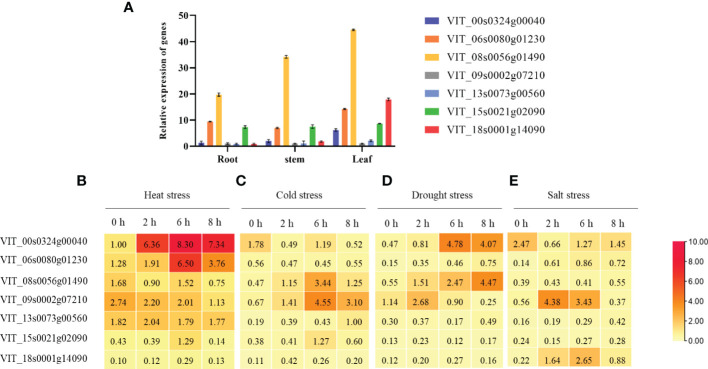
Different expression patterns of tissue and reaction by abiotic stresses in grapevine. **(A)** Tissue expression in root, stem and leaf. After heat stress **(B)**, cold stress **(C)**, drought stress **(D)** and salt stress **(E)** for 0 h, 2 h, 6 h and 8 h, the different expression profiles in grapevine. The results perform the mean ± SE of three independent replicates (*P* < 0.05).

To explore the functions of the 7 heat shock binding proteins detected, we examined their expression profiles in three grapevine tissues, and the results showed that they displayed different expression patterns in roots, stems and leaves (**Figure 4A**). For abiotic stresses, all of the heat shock binding proteins could be induced to different degrees (**Figures 4B–E**). First, these 7 heat shock binding proteins have different expression patterns during abiotic stress. VIT_06s0080g01230 only responded to heat stress (**Figure 4B**) but was not induced by cold stress, drought stress or salt stress. VIT_08s0056g01490 participated in cold and drought stresses (**Figures 4C, D**). VIT_09s0002g07210 was involved in cold and salt stresses (**Figures 4C, E**). However, VIT_13s0073g00560, VIT_15s0021g02090 and VIT_18s0001g14090 showed no obvious changes under abiotic stress. Significantly, VIT_00s0324g00040 could be induced by heat and cold stresses, especially 6 h of heat stress treatment (**Figure 4B**), which indicates that VIT_00s0324g00040 may play an important role in the response to heat stress in grapevine.

**Figure 5:**
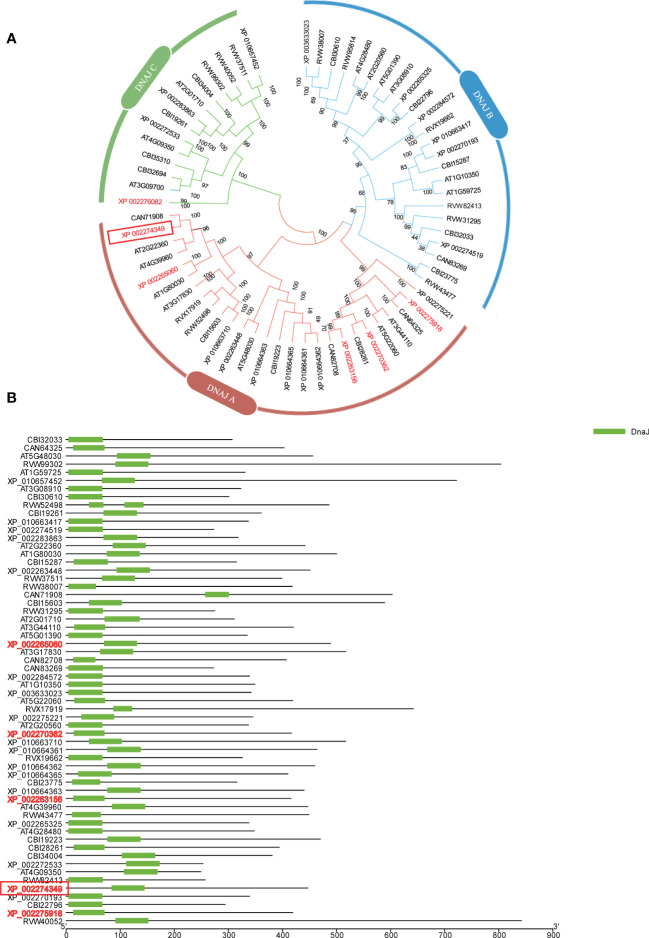
Bioinformatics Analysis with DNA JA6 protein. **(A)** Evolutionary tree analysis. **(B)** Conservative domain analysis. All protein names are uniformly named with NCBI accession ID. VIT_00s0324g00040 counterparted as XP 002274349 and marked with a red box.

The amino acid sequence of VIT_00s0324g00040 was used to blast the Arabidopsis genome to investigate which protein families it belongs to, and an evolutionary tree analysis was constructed according to the protein blast results. VIT_00s0324g00040 belongs to the DNA JA subfamily protein, and it was named DNA JA6 (**Figure 5A**). We also found that this DNA J family protein had a conserved DNA J domain (**Figure 5B**).

### DNA JA6 may binds to heat shock protein 70 in grapevine

**Figure 6:**
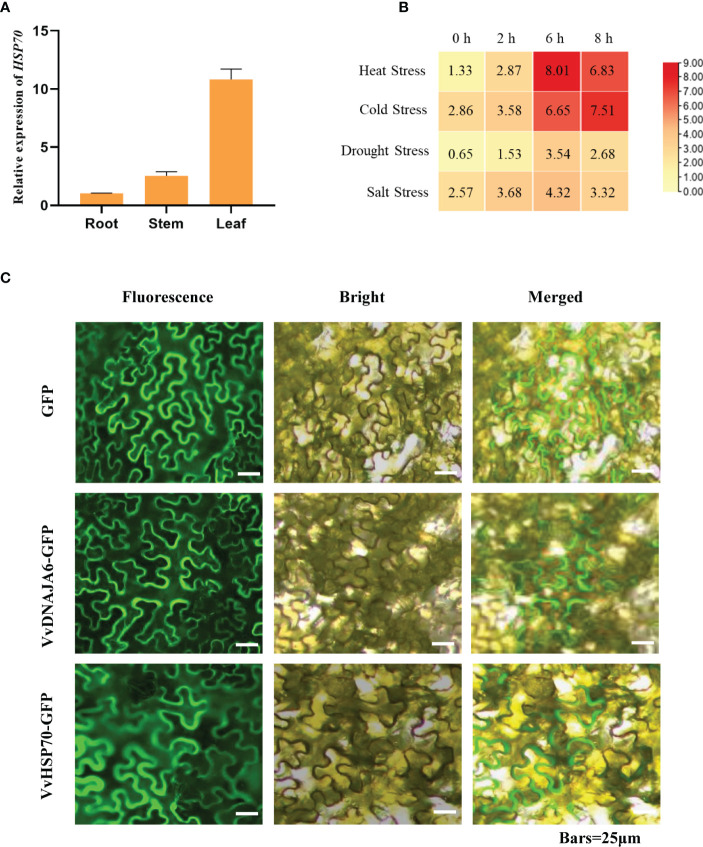
DNA JA6 may interact with HSP70 in Grapevine. **(A)** Relative expression of *HSP70* in root, stem and leaf. **(B)** Different expression patterns of *HSP70* in heat stress, cold stress, drought stress and salt stress. **(C)** Subcellular localization of DNA JA6 and HSP70. The results perform the mean ± SE of three independent replicates (*P* < 0.05).

DNA JA6, regarded as a cochaperone J-protein, builds on dependent protein refolding, which can enhance the interaction ability between HSP70 and subordinate proteins (Pulido et al., 2013; Craig and Marszalek, 2017; Jacob et al., 2017). First, organizational expression properties showed that *VvHSP70* was significantly highly expressed in grape leaves (**Figure 6A**). In addition, *VvHSP70* was significantly induced by cold and drought stresses, especially in response to heat stress (**Figure 6B**). Subcellular localization showed that both DNA JA6 and HSP70 were located on the plasma membrane (**Figure 6C**). Therefore, we speculate that DNA JA6 might bind to the HSP70-mediated heat stress response process synergistically in grapevine. But further yeast two hybrid result showed that DNA JA6 did not react with HSP70 (**Supplementary Figure 2**). This may be due to the interaction mechanism exists in Arabidopsis, but not in grapevine.

### DNA JA6 and HSP70 play a positive role in responding to heat stress

**Figure 7:**
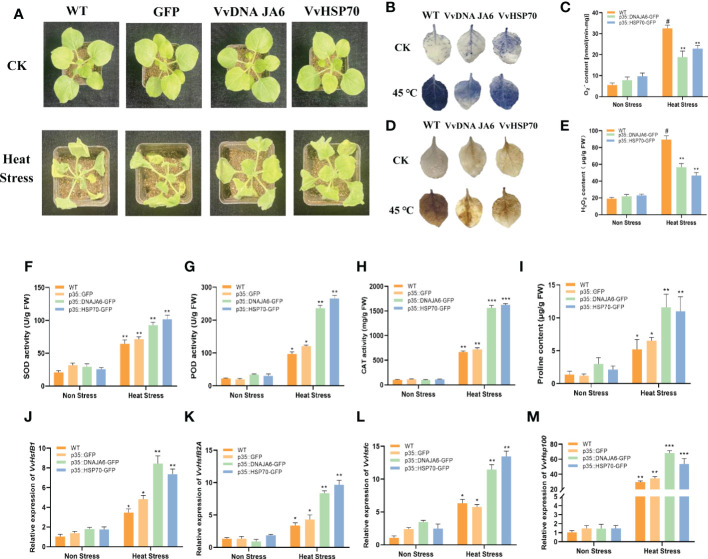
DNA JA6 and HSP70 play a positive role in response to heat stress. **(A)** The phenotype of WT, *DNA JA6* and *HSP70* transient over expression in tobacco after heat stress. The observation of NBT staining **(B)** and the test of O_2_^•-^content **(C)**. The observation of DAB staining **(D)** and the test of H_2_O_2_ content **(E)**. Analysis of physiological indexes SOD activity **(F)**, POD activity **(G)**, CAT activity **(H)** and proline content **(I)** in WT, *GFP*, *DNA JA6* and *HSP70* transient over expression tobacco after heat stress. Relative expression of heat stress marker gene *VvHsfB1* **(J)**, *VvHsfB2A* **(K)**, *VvHsfc* **(L)** and *VvHSP100* **(M)** in transient over expression tobacco after heat stress. The results perform the mean ± SE of three independent replicates (*P* < 0.05). “#” represent the object being compared. “*” represent difference. "**" represent significant difference. “***” represent extremely significant difference.

To further explore the significant function of DNA JA6 and HSP70 in responding to heat stress, an instantaneous transformation experiment was performed on tobacco to overexpress *VvDNA JA6* and *VvHSP70*. Under normal conditions, there was no obvious growth difference among the control (CK), *GFP* empty vector overexpression, and *VvDNA JA6* and *VvHSP70* overexpression lines. However, after heat stress, the growth of the *VvDNA JA6* and *VvHSP70* overexpression lines was significantly better than that of the CK and *GFP* overexpression lines (**Figure 7A**). The NBT staining results showed that the accumulation of dark blue compounds in tobacco leaves of wild type and VvDNA JA6 and VvHSP70 transiently overexpressed tobacco was similar, while compared with WT, the accumulation of transient overexpressed tobacco leaves was less after heat stress treatment (**Figure 7B**). The detection of O_2_^•-^ content showed that after treatment at 45 °C, the O_2_^•-^ content of wild type and transient overexpression tobacco increased, and VvDNA JA6 and VvHSP70 transient overexpression tobacco was lower than that of WT (**Figure 7C**). The results of DAB staining and content detection of H_2_O_2_ are similar to that of O_2_^•-^. After high temperature treatment, the accumulation of dark brown compounds (**Figure 7D**) and the content of H_2_O_2_ (**Figure 7E**) in tobacco leaves with VvDNA JA6 and VvHSP70 transient overexpression are less than WT.At the same time, overexpression of *VvDNA JA6* and *VvHSP70* in tobacco reduced the accumulation of MDA (**Supplementary Figure 3**) and increased the contents of the protective enzymes SOD (**Figure 7F**) and POD (**Figure 7G**) CAT (**Figure 7H**) and the osmotic substance proline (**Figure 7I**) to protect the plant during elevated temperatures. Moreover, compared with those in the CK and *GFP* overexpression lines, the transcripts of the high-temperature marker genes *VvHsfB1*, *VvHsfB2A*, *VvHsfC* and *VvHSP100* accumulated to a higher level in *VvDNA JA6-*and *VvHSP70*-overexpressing tobacco (**Figures 7G–M**). Altogether, these results indicate that *VvDNA JA6* and *VvHSP70* play a positive role in heat stress resistance enhancement in grapevine.

## Discussion

Proteins, as important materials for all life activities, exist in all cells (Sun et al., 2021). They are closely related to the growth and development of plants at various stages (Ling et al., 2012; Ling and Jarvis, 2015; Thomson et al., 2020). At present, increasing research on proteomics has provided new insights into the crucial functions of proteins in signal transduction, metabolism, and abiotic stress processes (Righetti and Boschetti, 2020; Smolikova et al., 2020). However, the essence of protein expression mainly involves the one-way flow from transcription to translation (Frohlich and Lindermayr, 2011). To further understand the interaction between the transcriptome and proteome, real-time monitoring of RNA and protein expression is required (Schaller et al., 2012). Therefore, we screened a class of molecular chaperone proteins by Ribo-seq and a series of bioinformatic methods, with the aim of investigating the complicated VvDNA JA6 and VvHSP70 mediated mechanisms induced by heat stress in grapevine.

Ribosome imprinting mapping provided new insights into gene expression on a translational level (Zoschke et al., 2013). Systematic translation analysis on different tissues of *Arabidopsis*, rice and maize, revealed many new translation sites and provided supplementary annotation of the genome (Zhu et al., 2021). In this work, 8291 proteins could be identified by using Ribo-seq in grapevine, and the translation sites were distributed in coding, UTR, intron and intergenic regions (**Figures 1A–C**). According to a previous report, when the ribosome moves across the new peptide chain, the codon will fluctuate by a frequency of 3 nt at the P-site (Bazin et al., 2017), which is consistent with the results of this study (**Figures 1D, E**). Then, we determined that proteins are heavily engaged in cellular metabolism and transportation using GO secondary function analysis. The majority of proteins exhibit kinase, transporter and antioxidant activities. Surprisingly, we discovered 7 heat shock binding proteins that react to heat stress (**Figure 2**). In addition, the RPKM value can be used as a criterion for translation efficiency. In the maize *tpj* mutant, the RPKM values of the *psbJ* and *psbN* genes were significantly lower than those of other chloroplast-related genes. The loss of the ribosome binding to *psbJ* and *psbN* ORFs by screen captures from IGV was also observed in the maize genome (Williams Carrier et al., 2019). In this study, different RPKM values implied different translational efficiencies for these 7 proteins (**Figure 3H**), and read abundance was visualized through IGV (**Figures 3A–G**). Further analysis of the evolutionary tree showed that the identified proteins belong to the DNA JA subfamily (**Figure 5A**). DNA J is an important chaperone protein in plants and is primarily classified into three subfamilies (DNA JA, DNA JB and DNA JC), which are composed of approximately 70 amino acids (Tamadaddi et al., 2021). The core structure is the His/Pro/Asp tripeptide (Craig et al., 2006). However, these proteins can also be divided into three types according to their structures. Type one contains J, G/P and CRR domains; type two contains J and G/P domains; and type three only contains J domains (Miernyk, 2001). In this work, the protein we screened for VvDNA JA6 only has a conserved J domain (**Figure 5B**). In addition, computer simulation analysis showed that a total of 120 DAN J proteins in *Arabidopsis* were distributed in different subcellular structures (Solana et al., 2022). 50 were located in the cytosol, 19 were located in the mitochondria, 12 were located in the chloroplast, 9 were located in the endoplasmic reticulum, 3 were located in the cytoskeleton, 1 was located in the plasma membrane, 2 were located in the vacuole, and 24 were located in the nucleus (Rajan and D'Silva, 2009). It has been reported that HSP70, another molecular chaperone, can interact with the J domain of DNA JA6 (Fernandez and Valpuesta, 2018), thereby regulating the activity of ATPase and maintaining the stability of protein complexes that participate in many biological processes (Usman et al., 2017). We found that although both VvHSP70 and VvDNA JA6 were located on the cell membrane, but VvHSP70 did not react with VvDNA JA6. So we speculate that there are many members of HSP protein family, and DNA JA6 heat shock binding protein may interact with other HSP protein families. (**Figure 6C**, **Supplementary Figure 3**).

Heat stress is an important issue since it causes aberrant biological processes, including protein polymerization and the release of some hazardous chemicals. When plant cells are damaged by heat stress, the cell membrane is initially affected by pressure initially, and the increased MDA content indicates that the plant has been seriously damaged. Then, heat stress causes a large amount of reactive oxygen species (ROS) to accumulate in plants. The J protein AtDjB1 protects cells by improving the activities of the antioxidant enzymes SOD, CAT and POD to counteract the heat-induced production of ROS in Arabidopsis (Zhou et al., 2012). HSP70 can combine with a molecular chaperone and is also affected by heat stress. Overexpression of *CaHSP70-2* also induced the expression of heat stress-related genes (*AtHsfA7a, AtHsp17.6C-CI* and *AtHsp25.3*) and improved the heat tolerance of Arabidopsis (Guo et al., 2016). In our work, when exposed to high-temperature stress at 45°C, compared with the wild type and GFP negative control, *VvDNA JA6* and *VvHSP70* transient transgenic tobacco showed a high-temperature tolerance phenotype (**Figure 7A**). Overexpression of *VvDNA JA6* and *VvHSP70* reduced the content of ROS (**Figures 7B–E**) and increased the activities of protective enzymes (SOD, PODand CAT) and the content of the osmotic substance proline (**Figures 7F, I**), as well as high-temperature marker genes (*VvHsfB1*, *VvHsfB2A*, *VvHsfC* and *VvHSP100*), under heat stress conditions (**Figures 7G–M**). These data proved that *VvDNA JA6* and *VvHSP70* play a positive role in heat stress resistance.

In summary, we propose that VvDNAJ A6 and VvHSP70 mediate the complicated mechanisms involved in increased grapevine thermoresistance, primarily by measuring a series of physiological indices and testing high-temperature marker genes relevant to the heat stress response. Thus, future work will verify the protein interaction between VvDNAJ A6 and VvHSPs family proteins in grapevine. Whether other chaperones interact with VvDNAJ A6 and VvHSP70 in the regulation of the heat stress response remains to be seen. Moreover, investigations into the control of the expression of core heat stress factors at the transcriptional level is needed. Since we observed read abundance data in the intergenic region, it is possible that noncoding RNA can be translated into protein.

## Conclusions

In this study, we used a novel technique called Ribo-seq to identify the whole protein expression profile of grapevine; we also visualized the translation of proteins and identified 7 heat shock binding proteins from the DNA J chaperone family. Of these seven proteins, VvDNAJ A6 was found to be highly upregulated by high temperature, to interact with VvHSP70, and to play a positive regulatory role in heat stress. Research regarding abiotic processes is critical for plant biology and for establishing the groundwork for identifying stress resistance proteins in grapevine.

## Data availability statement

The data presented in the study are deposited in the NCBI repository, accession number PRJNA903951.

## Author contributions

MC came up with the initial research; HB proposed some experimental strategies; ZZ performed experiments, interpreted data, and wrote the article. FD, SY, and ZL conducted the figure. LJ and HJ analyzed the data. All authors contributed to the article and approved the submitted version.
