# Supplementary material for: Translational profile of coding and non-coding RNAs revealed by genome wide profiling of ribosome footprints in grapevine
Source: Front Plant Sci. 2023 Feb 8;14:1097846. doi: 10.3389/fpls.2023.1097846 (PMC9944039; doi:10.3389/fpls.2023.1097846)
Supplement: Supplementary file 1 [file Table_1.docx]

Supplementary Material

## Supplementary Table 2

**Supplemental Table 2 Real-time PCR primers**

| Gene name | Primer sequence (5’ to 3’) |
| --- | --- |
| *NtACTIN* | FP:GAAGAAGGTCCCAAGGGTTC  RP:TCTCCCTTTAACACCAACGG |
| *NtHsfB1* | FP:ATCGTGGTGACAATGTGGCTAAT  RP:GCATCGCCTTCTGACTCATCTAA |
| *NtHsfB2A* | FP:GGAACAGGGTTTGTGGTATGG  RP:TGGAAGCCTTTAGGAATGGTG |
| *NtHsfc* | FP:TCCCAATAACAACATCATAGCACC  RP:GGATCAACCTTTCTGAAACCGTAG |
| *NtHSP100* | FP:AAGGGCATCATGGTGTTC  RP:TGTCCCTCAAGTCGTCAAG |

## Supplementary Figure 1


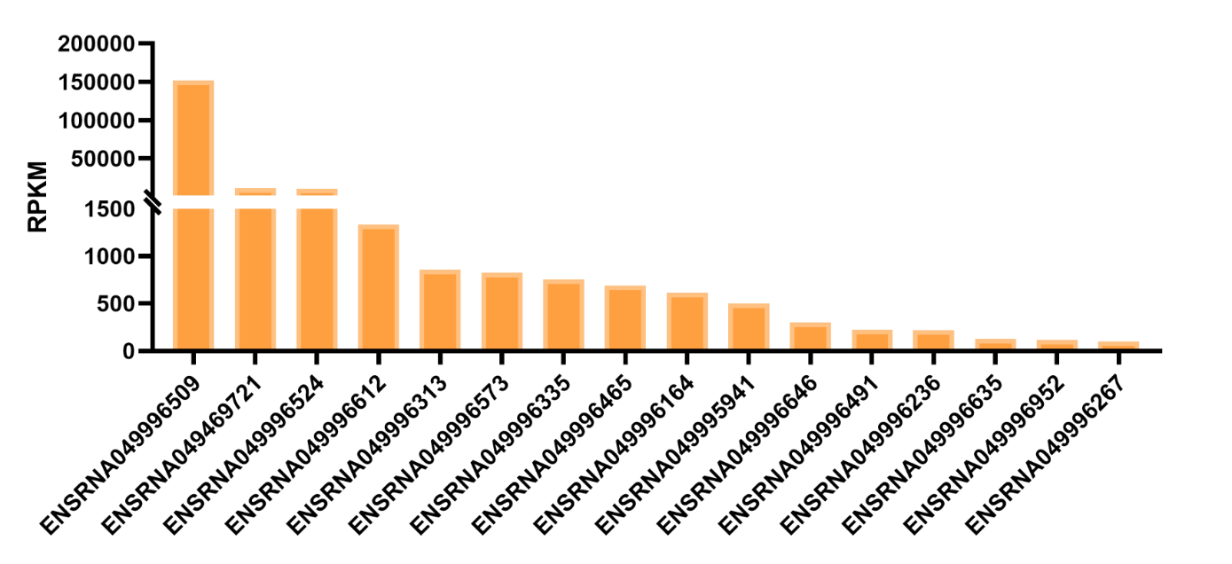

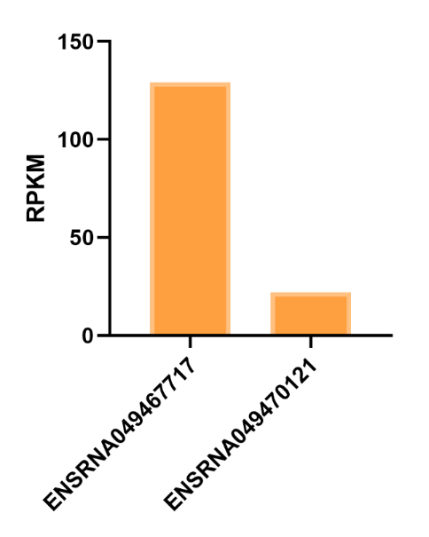

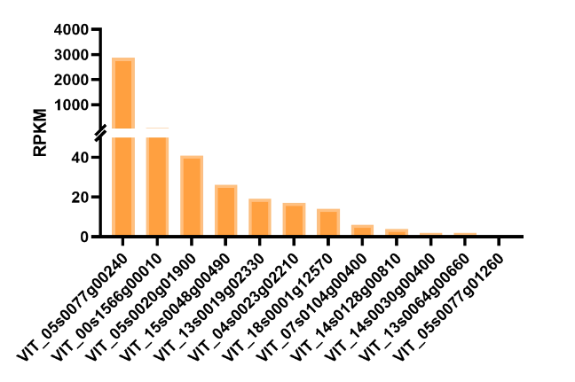


**A**

**B**

**C**

**Supplemental Fig. 1**

Supplementary Figure 1 |Different RPKM values of translated products falling in UTR (A), intron (B) and intergenic (C).

## Supplementary Figure 2


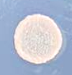

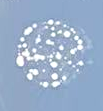

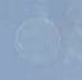

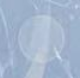

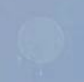

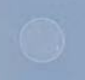

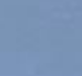

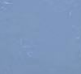


**1**

**1/10**

**1/100**

**AD-T+BD-53**

**AD-VvDNA JA6**

**+BD-VvHSP70**

**AD-T+BD-Lam**


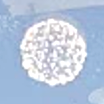


**SD/-Trp/-Leu/His/-Ade**

**Supplemental Fig. 2**

Supplementary Figure 2 |VvDNA JA6 protein does not interact with VvHSP70 protein

## Supplementary Figure 3


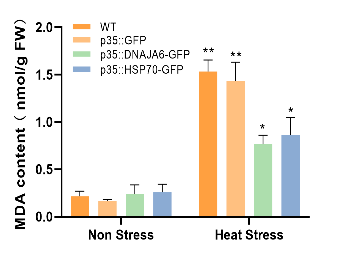


**Supplemental Fig. 3**

Supplementary Figure 3 |Analysis of physiological indexes MDA content.

**
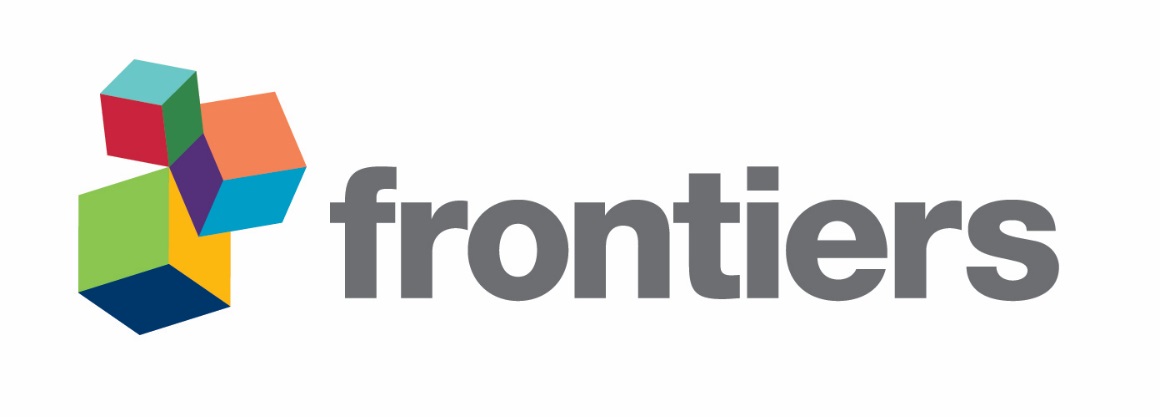
**
